# A recurrent missense variant in *HARS2* results in variable sensorineural hearing loss in three unrelated families

**DOI:** 10.1038/s10038-019-0706-1

**Published:** 2019-12-12

**Authors:** Leigh A. M. Demain, Erica. H. Gerkes, Richard J. H. Smith, Leslie P. Molina-Ramirez, Raymond T. O’Keefe, William G. Newman

**Affiliations:** 1 Division of Evolution and Genomic Sciences, School of Biological Sciences, Faculty of Biology, Medicine and Health, University of Manchester, Manchester, UK; 2 NW Genomic Laboratory hub, Manchester Centre for Genomic Medicine, St Mary’s Hospital, Manchester University NHS Foundation Trust, Manchester, UK; 3 Department of Genetics, University of Groningen, University Medical Center Groningen, Groningen, The Netherlands; 4 Molecular Otolaryngology and Renal Research Laboratories and the Department of Otolaryngology–Head and Neck Surgery, Carver College of Medicine, University of Iowa, Iowa City, IA USA

**Keywords:** Disease genetics, Neurological disorders

## Abstract

*HARS2* encodes mitochondrial histidyl-tRNA synthetase (HARS2), which links histidine to its cognate tRNA in the mitochondrial matrix. Biallelic variants in *HARS2* are associated with Perrault syndrome, a rare recessive condition characterized by sensorineural hearing loss in both sexes and primary ovarian insufficiency in 46,XX females. Some individuals with Perrault syndrome have a broader phenotypic spectrum with neurological features, including ataxia and peripheral neuropathy. Here, we report a recurrent variant in *HARS2* in association with sensorineural hearing loss. In affected individuals from three unrelated families, the variant *HARS2* c.1439G>A p.(Arg480His) is present as a heterozygous variant in trans to a putative pathogenic variant. The low prevalence of the allele *HARS2* c.1439G>A p.(Arg480His) in the general population and its presence in three families with hearing loss, confirm the pathogenicity of this variant and illustrate the presentation of Perrault syndrome as nonsyndromic hearing loss in males and prepubertal females.

## Introduction

*HARS2* encodes mitochondrial histidyl-tRNA synthetase (HARS2). HARS2 links histidine to its cognate tRNA in the mitochondrial matrix [1] and is an essential factor for mitochondrial translation [2]. Biallelic variants in *HARS2* have been associated with Perrault syndrome, a rare autosomal recessive disease characterised by variable degrees of sensorineural hearing loss (SNHL) in both sexes and primary ovarian insufficiency (POI) in 46, XX karyotype females [3, 4]. In some cases of Perrault syndrome additional neurological features, including peripheral neuropathy, cerebellar ataxia, and intellectual disability have also been identified [5]. Some individuals are reported to have white matter changes noted on magnetic resonance imaging of the brain [6]. To date, biallelic variants in six causative genes have been associated with Perrault syndrome:*HSD17B4* (MIM 233400) [7], *HARS2* (MIM 614926) [3], *LARS2* (MIM 615300) [8], *CLPP* (MIM 614129) [9], *C10orf2* (MIM 616138) [10], and *ERAL1* (MIM 607435) [6].

Previously, six unrelated families have been reported with variants in *HARS2* which cause Perrault syndrome. In a large family from North America, five affected individuals were compound heterozygous for the variants *HARS2* c.598C>G p.(Leu200Val) and c.1102G>T p.(Val368Leu). These variants reduced the aminoacylation activity of HARS2. All affected siblings had bilateral SNHL that varied in severity and age of onset. The three affected female siblings had ovarian dysgenesis and a 46,XX karyotype [3]. Two unrelated women with Perrault syndrome from consanguineous Moroccan families were homozygous for the same variant, *HARS2* c.1010A>G p.(Tyr337Cys). Both affected individuals are from the same region in Morocco and share a haplotype consistent with a founder variant. They have a similar phenotype of profound SNHL with onset before 3 years of age and secondary amenorrhea presenting at 25 and 26 years of age, respectively [11]. Three unrelated families with bialleic variants in *HARS2* were reported to all have rapidly progressive hearing loss [12]. In one family with SNHL, three affected individuals were compound heterozygous for the variants *HARS2* c.172A>G p.(Lys58Glu) and c.448C>T p.(Arg150Cys). Of note the two affected females in this family were 13 and 16 years of age, respectively. The proband from the second family, a 7-year-old female, was compound heterozygous for the variants *HARS2* c.448C>T p.(Arg150Cys) and c.980G>A p.(Arg327Gln). The third family comprised a 32-year-old female with Perrault syndrome and the variants *HARS2* c.137T>A p.(Leu46Gln) and c.259C>T p.(Arg87Cys) [12].

Here, we report three individuals with SNHL and previously unreported biallelic variants in *HARS2*. In each case, the affected individual is compound heterozygous for the variant *HARS2* c.1439G>A, p.(Arg480His) and a second putative pathogenic variant in *HARS2*.

## Materials and methods

Informed consent was obtained from all individual participants included in the study. All procedures performed in studies involving human participants were in accordance with the ethical standards of the institutional and/or national research committee and with the 1964 Helsinki declaration and its later amendments or comparable ethical standards. Ethical approval for this study was granted by the National Health Service Ethics Committee (16/WA/0017) and University of Manchester. Exome sequencing for individual F1-II-1 was performed by the Radboud University Medical Center, Nijmegen, The Netherlands. Before sequencing, genomic DNA fragments were enriched for exome sequences using the Agilent (Santa Clara, CA, USA) SureSelectXT Human All Exon 50 Mb kit. WES was performed at BGI-Europe (Copenhagen, Denmark), employing an Illumina HiSeq machine (Illumina, San Diego, CA, USA). Read alignment using the Burrows Wheeler algorithm and variant calling with GATK were performed at BGI. Variants were annotated with an in-house developed annotation and prioritization pipeline. Reported variants were only confirmed with Sanger sequencing in case of low quality (GATK quality scores) of the variant. Copy number variant calling was performed using CoNIFER 0.2.0, and variant annotation was performed using an in-house developed strategy. The median coverage was 97.5% with the HiSeq system. A panel of 142 hearing loss genes was analyzed [13]. Variants were classified according to the existing guidelines from the American College of Medical Genetics and Genomics [14]. The variants in families F2 and F3 were identified by hearing loss panel (OtoSCOPE^®^) at the Molecular Otolaryngology and Renal Research Laboratories at the University of Iowa, a Clinical Laboratory Improvement Amendments accredited laboratory. OtoSCOPE^®^ is a custom next-generation sequencing panel. Variants are mapped and analyzed using a custom pipeline before being confirmed by Sanger Sequencing. OtoSCOPE^®^ has a diagnostic sensitivity and specificity of >99% [15] All variants were mapped to the transcript NM_012208.3. Prediction of variant pathogenicity was performed using the following online resources: gnomAD (http://gnomad.broadinstitute.org/) [16], MutationTaster (http://www.mutationtaster.org/) [17], PolyPhen-2 (http://genetics.bwh.harvard.edu/pph2) [18], SIFT (http://sift.jcvi.org/) [19], ClustalOmega (https://www.ebi.ac.uk/Tools/msa/clustalo/). The variants have been submitted to the ClinVar database (https://www.ncbi.nlm.nih.gov/clinvar/), accession numbers; SCV000924703, SCV000924704, SCV000924705, and SCV000924706.

## Results

### Clinical reports

**Fig. 1:**
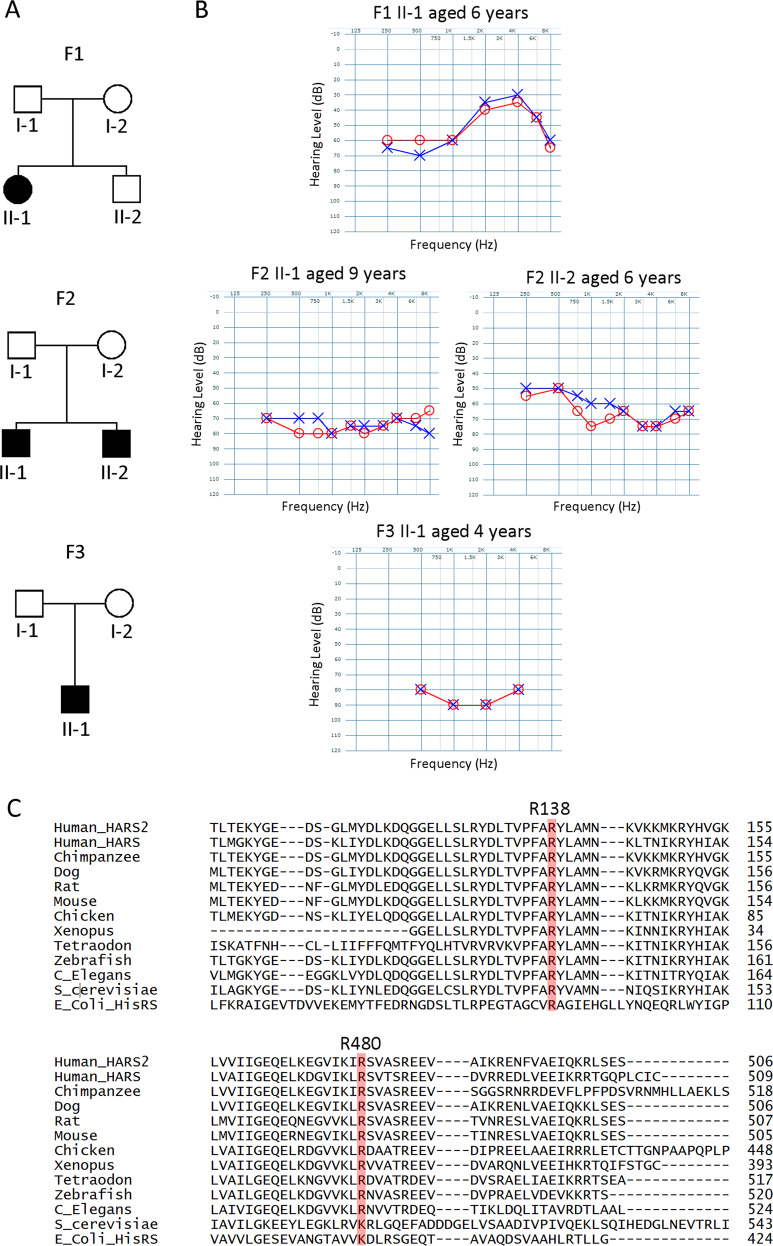
Pedigree, air conduction thresholds, and protein sequence alignment in individuals with *HARS2* variants listed in Table 1. **a** Pedigrees for families F1–F3. Filled icons indicate affected individuals. **b** Audiograms from affected individuals in families F1, F2, and F3. All affected individuals have bilateral sensorineural across all frequencies. Proband F1-II-1 shows lower air conduction thresholds in lower frequencies. Circles represent the right ear and crosses the left ear, dB decibels. Audiograms were created using the AudGen online tool (version 0.71) (http://audsim.com/audgen/). **c** The conservation of histidyl-tRNA synthetase across multiple species. The variant residues p.Arg138 and p.Arg480 are shown in the red boxes. Numbering relates to the human HARS2 protein (GenPept: NP_036340.1). Sequences for each species are as follows; chimpanzee UniProt: A0A2I3RUB6, dog UniProt:F6XKV4, rat GenPept:NP_001014034.2, mouse GenPept:NP_542367.1, chicken UniProt:A0A1D5P330, xenopus UniProt:F7A0W8, zebrafish GenPept:NP_001289185.1, tetraodon UniProt: H3CFH8, *C. elegans* GenPept:NP_001023373.2, *S. cerevisiae* GenPept:NP_015358.1, and *E. coli* HisRS UniProt: P60906.

Family F1 is a nonconsanguineous family of European descent and comprises an affected female proband, an unaffected younger child and her unaffected parents (Fig. 1a). The proband was diagnosed with moderate bilateral SNHL at age 6 years and was fitted with hearing aids. Audiometric testing revealed a more pronounced level of hearing loss at low frequencies (Fig. 1b). An average pure tone threshold of 48.75 in both ears at frequencies 512Hz, 1, 2, and 4 kHz of 48.75 dB (HL) was reported. At last assessment, no additional clinical features were present in the proband (Table 1). No relevant family history of similar hearing loss was reported.

**Table 1:** Clinical details and genotypes of individuals with Perrault syndrome associated with variants in HARS2.

Individual ID	Family 1 from Pierce et al. [3]	Individual IV-1 from Lerat et al. [11]	Individual VI-1 from Lerat et al. [11]	Family 1 from Karstensen et al. [12]	Family 2 from Karstensen et al. [12]	Family 3 from Karstensen et al. [12]	F1-II-1 (this study)	F2-II-1 (this study)	F2-II-2 (this study)	F3-II-1 (this study)
Variant	c.598C>G p.(Leu200Val); c.1102G>T p.(Val368Leu)	Homozygous c.1010A>G p.(Tyr337Cys)	Homozygous c.1010A>G p.(Tyr337Cys)	c.172A>G p.(Lys58Glu); c.448C>T p.(Arg150Cys)	c.448C>T p.(Arg150Cys); c.980G>A p.(Arg327Gln)	c.137T>A p.(Leu46Gln); c.259C>T p.(Arg87Cys)	c.413G>A p.(Arg138His); c.1439G>A, p.(Arg480His)	c.828delTinsGTATCCCTAGTATTTCTACTA (p.Gly277TyrfsTer3); c.1439G>A, p.(Arg480His)	c.72C>A, p.Cys24Stop; c.1439G>A, p.(Arg480His)	
Reference	Pierce et al. [3]	Lerat et al. [11]		Karstensen et al. [12]			This report			
Ethnicity	Mixed European	Moroccan	Moroccan	European	European	European	European	North American	North American	North American
Consanguinity	No	Yes	Yes	No	No	No	No	No	No	No
Sex	M and F (5 affected)	F	F	M and F (3 affected)	F	F	F	M	M	M
Karyotype	46, XX for 3 affected females	NR	NR	NR	NR	NR	46, XX	N/A	N/A	N/A
Age (years) at last assessment	Variable (13–37)	NR	NR	Variable (18–13)	7	32	6	14	11	4
Sensorineural hearing loss										
Age at detection (years)	Variable (3–31)	<3	<3	Variable (5 years–3 months)	2	2.5	6	2.5	1	1.5
Degree of hearing loss	Variable severity	Profound	Profound	Moderate/severe	Moderate/severe	Moderate	Moderate	Severe/profound	Severe/profound	Profound
Course of hearing loss	Progressive	None	None	Rapidly progressive	Rapidly progressive	Rapidly progressive	None	Progressive	Progressive	None
Hearing habilitation	Not recorded	Not recorded	Not recorded	Bilateral CI	Bilateral CI	Bilateral CI	HA	CI + HA	CI + HA	Not recorded
Gonadal dysfunction										
POI	Ovarian dysgenesis, with amenorrhea and streak gonads	Secondary amenorrhea, 25 years at onset	Secondary amenorrhea, 26 years at onset	One affected female has irregular menses at 16 years of age	N/A	Secondary amenorrhea, cessation of menses at 24 years	N/A	N/A	N/A	N/A
Notes	Affected males are fertile	None	None	None	None	None	None	None	None	None
Neurological features	None reported	None reported	None reported	None reported	None reported	None reported	None reported	One male has very tight muscles in his lower limbs, one has fine motor issues		None
Additional features	None reported	Hypothyroidism	Hypothyroidism	None reported	None reported	None reported	None reported	None reported	None reported	None reported

*HA* hearing aid, *CI* cochlear implant, *POI* primary ovarian insufficiency, *F* female, *M* male *N/A* non-applicable

Compound heterozygous variants in *HARS2*; c.413G>A p.(Arg138His), inherited maternally, and c.1439G>A p.(Arg480His) (NM_012208.3), inherited paternally, were identified as the likely cause of SNHL in the proband. The younger unaffected sibling was wild type at both loci.

Family F2 is a nonconsanguineous family comprising two affected male siblings and their unaffected parents (Fig. 1a). Proband F2-II-1 presented with bilateral mild-to-moderate SNHL at age 2.5 years. A mean pure tone threshold of 56.25 and 58.75 dB (HL) for the left ear at speech frequencies was reported. Further audiometric testing at age 9 years revealed an average pure tone threshold of 77.5 dB (HL) for the right ear and 71.25 dB (HL) for the left ear at frequencies 512 Hz, 1, 2, and 4 kHz (Fig. 1b). In light of the hearing loss in the older sibling, audiological testing for the younger sibling was requested (F2-II-2). Mild-to-moderate bilateral SNHL was detected in this sibling at age 1. Further audiometric testing at age 6 years revealed a mean pure tone threshold of 60 dB (HL) for the right ear and 58.75 dB (HL) for the left ear at frequencies 512 Hz, 1, 2, and 4 kHz (Fig. 1b).

Due to progression of the hearing loss, both siblings were reported to have undergone unilateral cochlear implantation. Both affected siblings have soft neurological features, for example, one has difficulty with fine motor movements, and the other has tightness in the muscles of his lower limbs. Neurological phenotypes are a well-recognized feature of Perrault syndrome; however, there is no concordance in these features between the brothers. Both siblings have had problems with tooth growth (Table 1). The compound heterozygous variants *HARS2;* c.828delTinsGTATCCCTAGTATTTCTACTA p.(Gly277TyrfsTer3) and c.1439G>A, p.(Arg480His) (NM_012208.3) were identified as the likely cause of SNHL in the affected siblings.

Family F3 is a nonconsanguineous family comprising the male proband (F3-II-1) and his unaffected parents (Fig. 1a). Bilateral profound SNHL was detected in this proband at 23 months of age. Sedated auditory brainstem response reported a mean pure tone threshold of 85 dB (HL) for both ears (Fig. 1b). Of note during testing using reversed polarity air conduction click a reversal of the cochlear microphonic was seen in both ears, suggestive of auditory neuropathy. At his last assessment, the proband was 4 years and had no additional clinical features (Table 1). The proband is currently under assessment for cochlear implantation. The variants *HARS2;* c.72C>A, p.(Cys24Ter) and c.1439G>A p.(Arg480His) (NM_012208.3) were identified as the likely cause of SNHL in the proband.

### Variants and predicted consequences

Human HARS2 likely functions as a homodimer. It contains predicted domains for histidine binding, dimer interaction, and tRNA binding [20]. HARS2 (NP_036340.1) shares ~73% sequence homology with nuclear HARS (NP_002100.2), from which it primarily differs at the N-terminus, and shares ~23% sequence homology with the *E.coli* orthologue HisRS (P60906).

The variant HARS2 p.(Arg138His) in individual F1-II-1 is present as a heterozygous variant in eight individuals of 141,443 sequenced (minor allele frequency, 0.000028) in gnomAD and has never been seen as a homozygous variant. This low-carrier frequency is consistent with a variant causative of rare autosomal recessive disease. HARS2 p.(Arg138His) is also predicted to be deleterious by multiple in silico analysis tools. The residue HARS2 Arg138 is an almost invariant residue conserved in both human HARS and the *E.coli* orthologue HisRS (Fig. 1c). Arg138 is situated in the predicted dimer interface region of HARS2. In *E.coli*HisRS, residue Arg90 (equivalent to residue Arg138 in HARS2) is located in the dimer interface region of the protein. *E.coli* HisRS Arg90 forms salt bridges with residues Asp13 and Glu47 of the other monomer in the dimer complex. These interactions both facilitate dimer formation and shape the active site [20]. The residues equivalent to Asp13 and Glu47 in human HARS2 are also conserved (Asp65 and Glu99 respectively). The substitution of arginine to histidine at residue 138 may interfere with salt bridge formation and subsequently dimer interaction and active site confirmation of HARS2.

The second variant in individual F1-II-1 HARS2 p.(Arg480His) is reported as a heterozygous variant in 21 of 141,421 individuals (minor allele frequency 0.000074) in gnomAD and never as a homozygous variant. HARS2 p.(Arg480His) is also predicted to be deleterious by multiple in silico analysis tools. The residue Arg480 is located in the C-terminal domain of HARS2, which is predicted as important for tRNA recognition and binding [20]. Residue Arg480 is well conserved but not as strictly invariant as the residue Arg138 (Fig. 1c). The effect of the substitution of Arginine for histidine at residue 480 is unclear, but it may disrupt recognition or binding of mitochondrial tRNA^His^.

In the affected individuals in families F2 and F3 the variant p.(Arg480His) is in trans with a loss-of-function variant, p.(Gly277TyrfsTer3) and p.(Cys24Ter), respectively. The transcripts for both of the loss-of-function variants are predicted to be subjected to nonsense mediated decay. Neither variant was present in gnomAD as either a heterozygous or homozygous variant. No homozygous loss-of-function variants are reported in *HARS2* in gnomAD.

## Discussion

Here, we report the variant *HARS2* c.1439G>A p.(Arg480His) in three unrelated families with prelingual onset, bilateral symmetric progressive SNHL. In each family the variant was in trans to a second putative pathogenic variant in *HARS2*. The identification of this rare missense variant in three unrelated individuals with SNHL means that it can be classified as moderate evidence of pathogenicity according to the American College of Medical Genetics and Genomics guidance on variant pathogenicity [14]. The report of these families increases the number of described families with *HARS2* variants and either Perrault syndrome or SNHL to nine.

In the proband from family F1 the variant in trans to HARS2 p.Arg480His may interrupt the dimerization and active site confirmation of the HARS2 homodimer. Given the conservation and function of p.Arg138 as well as the in silico predictions and the minor allele frequency it is likely that the substitution p.Arg138His is deleterious. In affected individuals from families F2 and F3 the variant p.(Arg480His) is in trans to a loss-of-function variant. No cases of biallelic loss-of-function variants in any of the six genes associated with Perrault syndrome have been reported to date [21]. This lack of biallelic loss-of-function variants likely indicates that *HARS2*, along with the other Perrault syndrome associated genes, is essential and that complete loss-of-function would result in lethality. Of note, the loss of the HARS2 yeast orthologue, HST1, is lethal [22]. If homozygous loss of HARS2 is lethal only combinations of hypomorphic alleles in trans with loss-of-function alleles, or biallelic hypomorphic alleles can be expected to result in a phenotype.

Of note, the hearing loss in the individual with two missense (putative hypomorphic) *HARS2* variants is milder (especially at higher frequencies) than in the other two families, where a missense (hypomorphic) variant is in trans with a predicted loss-of-function variant. Further follow-up of these individuals and ascertainment of additional cases will determine if this genotype–phenotype association represents a robust observation.

Currently, there appears to be no link between the variants in *HARS2* reported to be causative of Perrault syndrome and their location in the HARS2 protein. The reported pathogenic variants in HARS2 are located in multiple domains with no specific domain linked to dysfunction. In contrast, variants in *CLPP* linked to Perrault syndrome are clustered mainly in a single 20 residue region of the CLPP protein [21].

In the families reported here a diagnosis of SNHL revealed variants in a Perrault syndrome related gene, which would not have been suspected based on clinical presentation alone. All affected individuals are either male or prepubertal and as such would not present with POI, a cardinal feature of Perrault syndrome. In the case of family F1 the female proband is prepubertal and will be monitored for POI. It is possible that hypomorphic variants in Perrault syndrome genes may cause milder presentation of hearing loss or POI only and therefore the individuals from F1 or F3 may have nonsyndromic SNHL [21]. It is less likely that the neurological features reported in family F2 can be attributed to the *HARS2* variants as they are not concordant in the affected siblings. Further follow-up and evaluation of additional individuals with *HARS2* pathogenic variants will be required to establish any neurological phenotype. Neurological presentations are commonly associated with Perrault syndrome and although no phenotype genotype links have yet been established for *HARS2*, all individuals with disease associated *HARS2* variants should have a full clinical neurological assessment. As panel and exome testing becomes more commonplace for etiological diagnosis in hearing loss, we expect more cases of *HARS2*-associated Perrault syndrome will be recognized. Timely confirmation of etiological diagnosis and subsequent prediction of profound severity in individuals with *HARS2* variants could help inform the identification of cases that may benefit from referral to cochlear implantation services and closer audiological surveillance [23].
